# Effects of Microvibrations and Their Damping on the Evolution of Pinot Noir Wine during Bottle Storage

**DOI:** 10.3390/foods11182761

**Published:** 2022-09-08

**Authors:** Simone Poggesi, Vakarė Merkytė, Edoardo Longo, Emanuele Boselli

**Affiliations:** 1 Oenolab, NOITechPark Alto Adige/Südtirol, Via A. Volta 13B, 39100 Bolzano, Italy; 2 Faculty of Science and Technology, Free University of Bozen-Bolzano, Piazza Università 5, 39100 Bolzano, Italy

**Keywords:** natural magnetic levitation, wine storage, microvibrations, quantitative descriptive analysis, GC × GC–MS, LC–MS, multivariate statistics of wine

## Abstract

Environmental conditions such as vibrations, temperature, and exposure to light can lower the quality of bottled wine, causing great economic and image losses for wineries. Even under optimal storage conditions, environmental microvibrations can be a constant source of energy transfer to the stored bottles, and little is known about their effects over time. In this study, the effects of microvibrations on a fine Pinot noir wine were evaluated over a storage period of one year under controlled conditions and compared with those obtained using natural magnetic levitation as a damping technique to reduce the power transmitted by the vibrations. The wines were subjected to the treatments according to the following experimental set-up: (A) wines not exposed to microvibrations, but to natural magnetic levitation; (B) wines placed on a shelf in contact with the floor, and exposed to microvibrations; (C) controls, a shelf in direct contact with the floor, without the application of microvibrations; (D) wines on a shelf with natural magnetic levitation and exposed to microvibrations. Phenolic and volatile compounds were not significantly different between treatments, which is in line with the reduced energy stress applied. In contrast, the storage time significantly influenced these chemical profiles. Through the sensory analysis performed after 0 and 12 months of storage, it was possible to distinguish the wines, as the overall quality improved, especially for the microvibration-treated samples. After 12 months of storage: (a) the overall sensory quality improved for all wines compared to the samples at T0; (b) the damping of microvibrations reduced the rate of wine evolution; (c) treatment with microvibration up to 6 months was useful for improving the quality of wine not yet ready for the market. Therefore, modulation of wine evolution can be achieved by applying a combination of microvibrations and their damping, depending on the enological objective.

## 1. Introduction

Wine bottling is the last winemaking step in which the winemaker can make changes to the product to influence its quality. Therefore, from bottling to drinking, it is important to keep the bottled wine in the best possible environmental conditions, in order to preserve quality. Several factors may influence the quality of bottled wines, such as light exposure, temperature, oxygen transfer, and vibration. In previous literature, the concept of chemical age was investigated by considering the metabolic change during ageing of twenty Sangiovese wines stored for 24 months [1]. The authors also defined three stages of wine lifetime: the maturation, in which taste, flavour, and wine stability are improving; the maturity stage, in which the wine is at the peak of its quality; a third stage, in which the wine quality decreases [2]. The effects of temperature and vibrations on wines transported by trucks within Europe were also monitored [3]. These studies showed that the influence of the journey on the wine ageing was dependent on the wine matrix. Additionally, the combination of vibrations and movements at a high temperature (40 °C) led to lower wine quality. Moreover, if the environmental conditions are not adequate, wines can experience pinking or browning, and sensory modifications, with a decrease in the fresh and fruity aromas. The effect of transportation and vibrations on white wines can lead to some problematic changes in sensory attributes: the wines that underwent high levels of vibrations had lower concentrations of propanol and isoamyl alcohol, which are important for the freshness and fruity aromas [4]. Furthermore, they reported that vibrations of 2–5 Hz can be dangerous for fragile products, such as glass bottles, unless they are packed in suitable and safe boxes. Different levels of vibrations applied to wines (1, 5, 10 Gal, cm s^−2^) for 18 months produced no effects, in contrast to a decrease in quality observed when wine was subjected to vibrations of 20 Gal [5]. The effects of vibrations can increase during wine transportation, and it is important to control the environmental conditions during the storage and transport phases, especially for premium wines that are usually aged for decades [6,7,8]. Despite the wealth of information on the direct effect of vibrations and temperature, or of combinations thereof, on wine quality, little is known about the effects of natural environmental microvibrations on stored bottled wines. These vibrations can be present even under the best applicable storage conditions. However, to study their effect, the source of these vibrations must also be removed.

Therefore, this work aimed to study the effects of microvibrations from the environment over a 12-month storage period on high-quality bottled wine maintained under optimal storage conditions (stable environmental temperature, no source of high-energy vibrations, no light sources, and screw-cap closures to ensure no oxygen transfers). The samples subjected to microvibrations were compared to bottles of the same wines placed on a vibration-damping shelf (obtained through repelling natural magnets), which reduced the vibrations from the environment by converting disordered mechanical vibrations into a harmonic wave. Overall, the samples were subjected to four different treatments: (A), wines at ambient condition with applied natural magnetic levitation and no applied microvibrations; (C), (control), wines on a shelf in direct contact with the floor; (D) wines subjected to microvibrations but placed on a shelf with applied natural magnetic levitation; and (B) wines subjected to microvibrations. Chemical (volatile and phenolic compounds) and sensory parameters were monitored during the 12-month storage period.

## 2. Materials and Methods

### 2.1. Materials and Chemicals

A local winery (Franz Haas, Montagna, Italy) supplied the Pinot Noir premium bottles, all closed with screw caps, that were obtained from a unique batch. For the analysis, all chemical standards, solvents, and mobile phase modifiers were purchased from Merck Life Science S.r.l. (Milano, Italy). Ultrapure water was obtained using a AriumMini generator (Sartorius Italy S.r.l., Varedo, Torino, Italy). The purchased mobile phases and related modifiers (formic acid) were of LC-MS grade.

### 2.2. Wine Samples and Vibration Damping Devices

The wines were placed on shelves built-in 4 different configurations (Wineleven, Appiano S.d.V., Italy). The microvibration damping device was a shelf formed by two separate rectangular-shaped elements; the top element could host up to 18 bottles in a horizontal position, whereas the bottom element could be laid on the floor; the two elements were separated by an air cushion formed by repelling magnets positioned on the surface of the four corners of the two elements. Repelling magnets form a device which resists motion via viscous friction. The resulting force is proportional to the velocity, but acts in the opposite direction, slowing the motion and adsorbing the energy [9,10,11]. Natural repelling magnets use magnetism to transform non-harmonic vibrations into harmonic vibrations and were used to reduce the destructive energy vibrations reaching the bottle.

**Figure 1:**
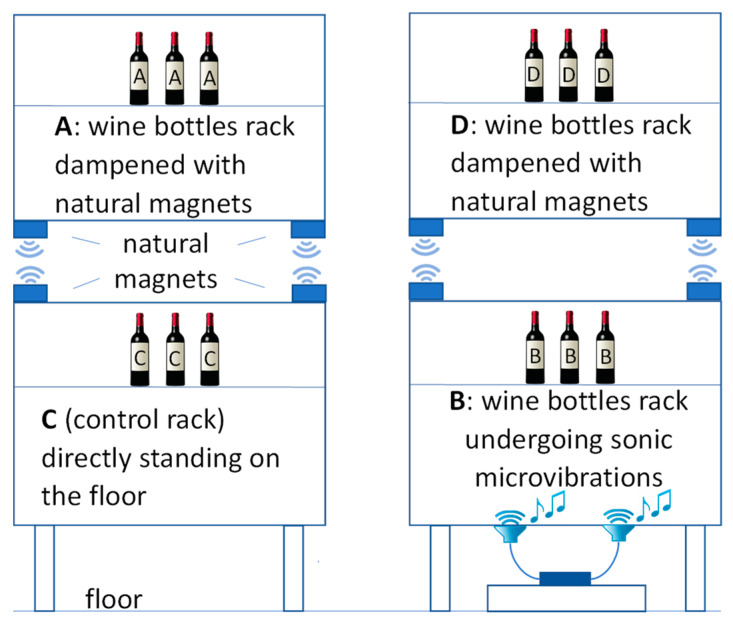
Configuration of the shelves hosting the wine bottles during the storage. The image shows the bottles for the two shelves used in the study.

Figure 1 shows the different configurations of the shelves equipped with/without sonic microvibrations and natural repelling magnets: A, environmental conditions with natural magnetic levitation; C, (control), a shelf in direct contact with the floor; D, shelf with natural magnetic levitation, and B, a shelf in contact with the floor and equipped with a microvibration system (a sound diffusion system—consisting of two low-frequency woofers and an amplifier system with a digital track readout for low frequency emission (60–250 Hz) and low decibels (the diffusion system was set up to constantly play a series of sounds of very low frequency, in order to create microvibrations on the shelves). The two shelves were kept in a standard environmental controlled temperature conditions (average at 20 °C and relative humidity between 50 to 70%). Therefore, the changes were due to the ageing and vibration of the bottles. The samples were analysed in duplicate at the beginning of the study (T0), after 1 month (T1), after 3 months (T3), after 6 months (T6), and after 12 months (T12) of storage.

### 2.3. Phenolic Profile by HPLC-DAD/FLD and LC-QqQ-MS

The phenolic compounds were analysed by HPLC-DAD/FLD, as described by Dupas de Matos, et al. (2020) [12], and offline HPLC-ESI(−)/QqQ/MS for the tentative identification of compounds. Briefly, a Eurosphere II Knauer (250 mm × 4.6 mm × 5 μm, C18) from Lab Service Analytica, Bologna, Italy) with a C18 stationary phase was used, and mounted on a Nexera X2 UHPLC system (Shimadzu, Milano, Italy) equipped with a UV-Vis diode array (DAD) and a fluorescence detector. The HPLC mobile phase was solvent A (0.1% of LC–MS grade formic acid purchased from Merck Life Science S.r.l., Milano, Italy, in ultrapure water) and solvent B (0.1% formic acids in acetonitrile, MS grade). The gradient separation program was as follows: 1% B from 0 to 2.5 min; 1 to 25% B from 2.5 to 50 min; then 25% to 99% B in 1 min; 99% B from 51 to 55 min; back to 1% B from 55 to 58 min; and final reconditioning at 1% B phase from 58 to 60 min. The flow rate was 0.7 mL/min. The peak integration was performed automatically by the software provided by Shimadzu (Labsolution) and the peak alignments were performed manually. Besides, unknown compound assignment was performed offline on an LC-DAD-ESI/QqQ/MS in negative mode (Agilent, Santa Clara, CA, USA) applying the same separation conditions and ESI- ionization mode. Table S1 shows the tentative compound identification by offline HPLC-DAD-ESI/QqQ/MS in negative mode [13,14,15,16,17].

### 2.4. Profile of Volatile Compounds with Headspace Microextraction Bidimensional Gas-Chromatography Mass Spectrometry

The volatile profile has been characterized by headspace solid-phase microextraction–bidimensional gas chromatography coupled with time-of-flight mass spectrometry (HS-SPME GC × GC-ToF/MS, (LECO Italy S.r.l., Cassina de’ Pecchi, Milano, Italy). For each wine sample, 4 mL was placed in a 10 mL vial along with 500 mg of NaCl and 5 μL of a stock solution of internal standard (100 μL of 3-methyl-2-pentanol—purchased from Merk Life Science S.r.l., Milano, Italy—in 10 mL of ethanol). For the analysis, each sample was incubated with 300 rpm stirring at 40 °C for 15 min. Then, a pre-conditioned triphasic SPME fibre (50/30 µm DVB/CAR/PDMS, Stableflex, 23 Ga, 1 cm—Sigma-Aldrich, St. Louis, MO, USA) was inserted into the sample headspace. Then, the sample was injected for the analysis using an autosampler: the injection time was 6 min and the GC inlet was set at 240 °C. In the GC × GC gas chromatograph, the first dimension was a polar MEGA-WAX spirit column (PEG phase) 40 m/0.18 mm/0.30 μm, and the second column was a MEGA 1-HT 1.2 m/0.1 mm/0.1 μm (MEGA, Milano, Italy). The flow modulator was a FLUX (Leco, Geleen, The Netherlands) that uses helium to modulate the passage of the eluent and the analytes from the first to the second dimension; the modulator parameters were set as a modulation period of 2.5 s and an injection time of 0.08 s. The fibre injection at the GC × GC front inlet lasted 6 min at 240 °C, in spitless mode; the carrier gas flow rate applied was 1 mL/min. The separation was performed at a constant flow rate. The temperature ramp of the main GC oven was 40 °C for 6 min (injection), then from 40 °C to 180 °C at 3 °C/min, then from 180 °C to 240 °C at 10 °C/min, and 1 min at 240 °C. The secondary oven was kept constantly at +5 °C above the primary oven. Detection was performed on a pre-tuned ToF detector, according to the following parameters: solvent delay, 0 min; acquisition rate, 150 spectra/sec; acquisition mass, 35–650; extraction frequency, 32 kHz. The processing software ChromaToF^®^ (LECO Corporation, Berlin, Germany) ver. 2021, was used to process the chromatograms obtained from bidimensional gas chromatography and a tentative compounds assignation was performed using NIST library 2017 (NIST MS search 2.3). Table S2 reports the tentative identification of volatile compounds based on the retention time in the first and second dimension analysed by GC × GC-TOF/MS.

### 2.5. Sensory Profile

Thirteen students and technical staff (7 females and 6 males, 29 ± 7 years old) were recruited from the Free University of Bolzano-Bozen (Italy) based on interest and availability. All panellists voluntarily agreed to participate and signed an informed consent. The panellists had no prior experience with formal sensory evaluation and particularly with Quantitative Descriptive Analysis (QDA^®^). The panel performed 7 initial sessions (1 h each), during which each panellist was introduced to sensory protocols and received instructions from the panel leader on how to recognize and evaluate the sensory descriptors. The initial phase focused on defining the descriptors to be used in the subsequent sensory analysis. In this qualitative phase, the assessors were asked to choose among some sensory descriptors based on two reference wines (both produced in South Tyrol). The final common vocabulary is reported in Table 1. It consists of three visual descriptors (clarity, colour tonality, and colour intensity), five aroma descriptors (red fruit, dried fruit, undergrowth, spicy, and woody), six taste descriptors (warmness, astringency, sourness, sweetness, bitterness, and saltness) and two flavours (red fruit and woody). Subsequently, the panellists undertook specific training in 6 different sessions (60 min for each, 2 sessions per week for 3 weeks) on the chosen descriptors. The sensory training was conducted following the ISO 8586:2021 protocol. Training of aroma descriptors was performed by asking panellists to identify different aromas such as strawberry, blueberries, raisin, prune, mushroom, clove, black pepper, star anise, liquorice, vanilla, oak, and coffee. Training on taste descriptors was also performed with grading test (9-point scale) based on the concentration of the samples. Shared taste descriptors were sweetness (0, 0.5, 2 g/L sucrose), bitterness (0, 1, 2 g/L caffeine), sourness (citric acid 0.5, 1, 2 g/L, and tartaric acid 0.5, 1, 2 g/L), astringency (0.5, 1, 2 g/L alum), and warmth (8, 12, 18% *v*/*v* alcohol). All standard solutions were prepared using food-grade reagents.

### 2.6. Statistical Analysis and Data Processing

The statistical analyses were performed using XLSTAT software (Addinsoft, New York, NY, USA). The whole dataset was divided into three sub-datasets: phenolic compounds, volatile compounds, and sensory data. Each dataset was elaborated using principal component analysis (PCA) by the correlation method and standardized by 17 samples. The dataset of sensory analysis was computed with one-way ANOVA on treatment and pairwise comparison with Tukey’s HSD and Duncan’s post hoc test. The confidence levels were set at 95% (critical limit of *p*-value < 0.05).

MFA (multifactor analysis) was applied as a data fusion method: the datasets were included as six separate tables to be integrated into a single projection over the multivariate partial axes created by the MFA. The first four tables contained sensory analysis variables, separated according to the method of perception (visual, olfactory, taste and flavours, and overall quality judgment), and the last two tables contained the volatile and phenolic profiles, respectively.

## 3. Results

### 3.1. Phenolic Compounds

**Figure 2:**
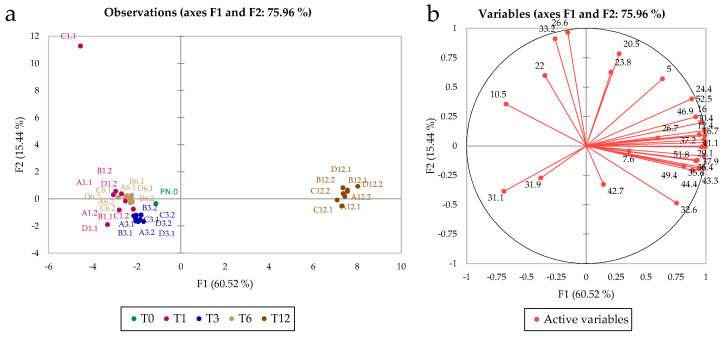
Observations and plots of variables based on HPLC-DAD data. Loadings are labelled by peak retention times. Numbers refer to months (of storage) and sample replicates. Letters refer to the specific treatment (see Figure 1 for details on the treatment). Please, see Table S1 (Supporting Information) for details on the peak assignment. (**a**) Scores plot, (**b**) Loadings plot.

Figure 2 shows the PCA plots based on the phenolic profiles of the samples. The model that included the first two principal components accounted for 83.5% of the total variance. Figure 2a shows the remarkable effect of storage time on the composition of the phenolic profile in the entire sample set; in fact, the model differentiated two groups of samples: Time 1 (after 30 days), T3, and T6 grouping together and T12 separated by PC1.

ANOVA showed that all variables were significantly different for storage time, but only two variables (x.12—*m*/*z* 579.1, and x.15, not identified) were significant for the treatment (Table S3). Tentative identification of the phenolic compounds is provided in the Supporting Information (Table S1).

### 3.2. Volatile Compounds

The volatile compounds analysed by GC × GC-TOF/MS were assigned using the automatic alignment performed by ChromaToF Tile application.

**Figure 3:**
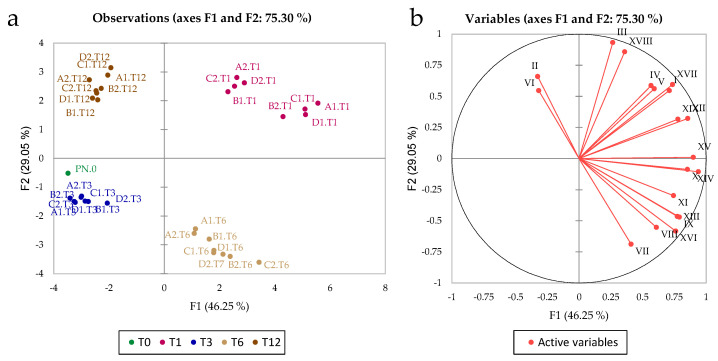
Observations and variables graph from GC × GC-TOF/MS data. T0, T1, T3, T6, and T12 indicate the storage months (0, 1, 3, 6, 12) at which the samples were analysed. A, B, C, and D indicate the treatment (see Figure 1 for details). (**a**) Scores plot, (**b**) Loadings plot.

Figure 3 shows the PCA model obtained from the bidimensional gas chromatography data; a high percentage (75.3%) of the total variance was explained by the two first components of PCA. Again, the clustering of the samples mainly reflected storage time; thus, four different clusters of samples can be observed. The variables II (ethyl acetate) and VI (1-hexanol) correlated with T12, whereas variables III (*n*-propyl acetate), XVIII (2,4-di-terbuthylphenol), IV, V (1-butanol, 3-methyl-), I (acetaldehyde), XVII (phenylethyl alcohol), XIX (hexadecenoic acid, ethyl ester), XIII (decanoic acid, ethyl ester), and XV (3-buten-2-one, 4-(2,6,6-trimethyl-1-cyclohexen-1-yl)) correlated with T1 and variables VII (4-amino-1-butanol), VIII (propanoic acid, 2-hydroxy-, ethyl ester), and XVI (butanedioic acid, diethyl ester) correlated with T6. T0 and T3 were anti-correlated to the variable that correlated with T1. ANOVA performed on the volatile compounds showed that all variables were significant in differentiating storage time, but did not differentiate treatment (Table S4).

### 3.3. Sensory Analysis

**Figure 4:**
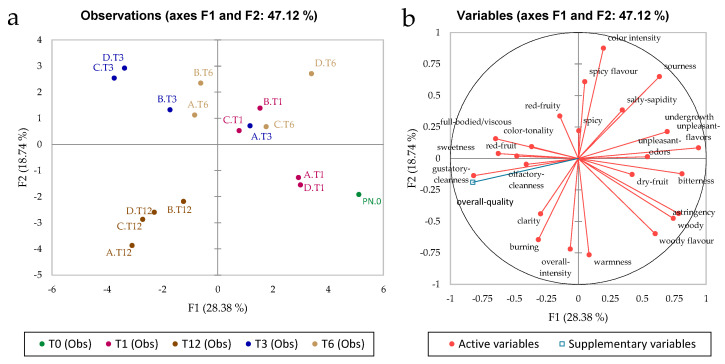
Plots of observations and variables constructed from the sensory data. T0, T1, T3, T6, and T12 indicate the storage month (0, 1, 3, 6, 12) at which the samples were analysed. A, B, C, and D indicate the treatment (see Figure 1 for details). (**a**) Scores plot, (**b**) Loadings plot.

Figure 4 shows PCA plots constructed on the sensory data of all samples; 47% of the total variance was explained by the first two principal components. The PCA model showed greater dispersion than those of phenolic and volatile compounds. There were two main clusters: the first at T12, which correlated with the overall quality judgment scores (OQJ: a score provided by the panel on the overall quality of the wine, which was included here as an additional variable projected in the PCA). T12 also correlated with “olfactory cleanness”, “gustatory cleanness”, “clarity”, “burning”, and “overall intensity”. It is worth noting that the samples stored on shelves dampened by natural magnetic levitation (treatment A.T12) are closest to the “overall intensity”.

Samples stored for shorter times (T0, T1, T3, and T6) formed a more diffuse cluster (in the remaining three quadrants of the diagram), showing a marked difference from T12 and greater heterogeneity. The T1 treatments were separated along PC2, and can be divided into two clusters, as treatments A and D correlated positively with “woody” aroma and flavour, “dry fruit” aroma, “bitterness” and “astringency”. In contrast, treatments B and C for T1 correlated with higher “spicy” aroma and flavour, “salty-sapidity”, “sourness”, and “colour intensity”. Time 3 treatments were separated along PC1, again into two clusters, the first of which comprised treatments B, C, and D, which were correlated with higher “full-body/viscosity”, “colour tonality”, “sweetness”, and “red fruit” aroma and flavour scores. In contrast, treatment A.T3 correlated with the same variables as B.T1 and C.T1 described above. The T6 wine samples were also divided into two main clusters: one cluster included samples A and B, which were located close to B, C, and D at Time 3 and the second cluster consisted of samples C and D, which were correlated with certain negative sensory descriptors, such as “sourness”, “undergrowth”, and “unpleasant” odours and flavours.

**Figure 5:**
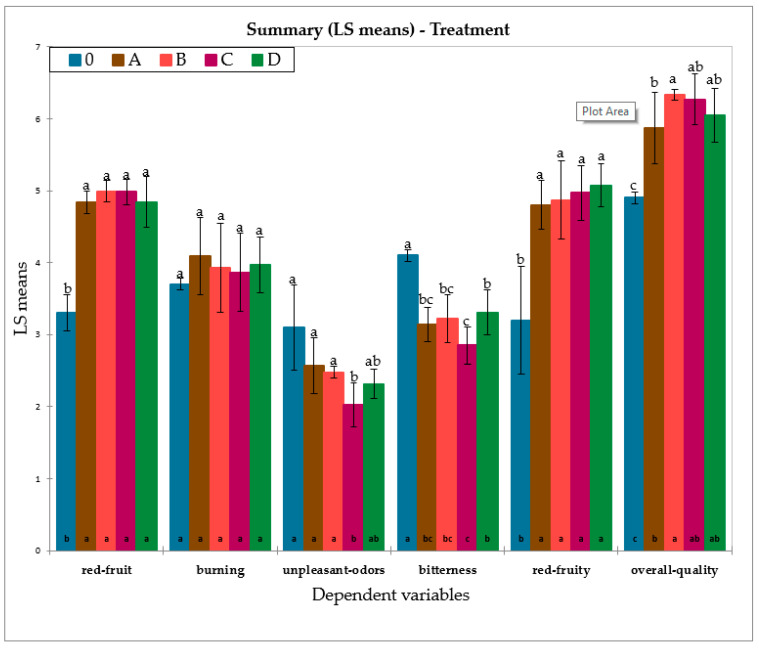
Bar chart plot for the significant variables in the one-way ANOVA with Duncan’s post hoc test. A, B, C, and D indicate the applied treatments (see Figure 1 for details). 0 indicates samples at time 0. Lower case letters (a, ab, b, bc, c) represent the computed groupings of the groups by the Duncan’s post hoc multiple range test.

**Figure 6:**
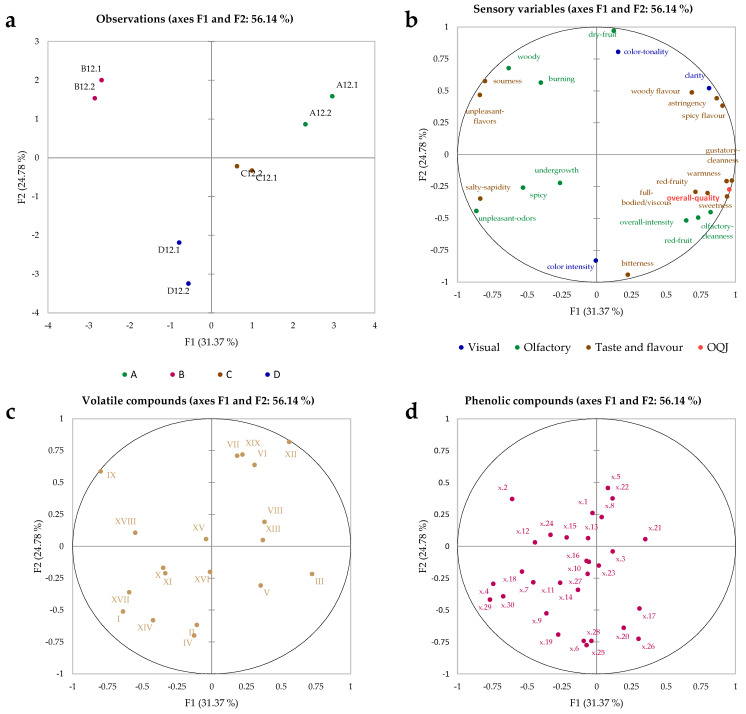
MFA plots on the T12 dataset: (**a**) observation plot, (**b**) sensory analysis variables plot, (**c**) volatile compounds variables plot, and (**d**) non-volatile phenolic compounds variables plot. T0, T1, T3, T6, and T12 indicate the storage month (0, 1, 3, 6, and 12) at which the samples were analysed. A, B, C, and D indicate the treatment (see Figure 1 for details).

The entire sensory dataset from T0 to T12 was analysed by means of ANOVA and Duncan’s post hoc test to see which variables were most influenced by the treatment. Figure 5 highlights the variables that significantly differentiated the treatments, which were: red fruit aroma, unpleasant odours, bitterness, red fruit flavour, and overall quality judgment. It should be noted that time 0 (T0) was completely different from the other times and treatments and was included to better highlight the overall effect of the treatment over a 12-month period compared to the initial sample. The overall quality score of the wine at T0 was lower than at all other times (the wine was not yet ready for market). Interestingly, the overall quality scores of the treatment with damping (treatment A) was more similar to the control (treatment C) than the other treatments, showing that vibration damping applied to the control samples reduced the rate of evolution of the wine. As Figure 5 shows, treatment B scored the highest for the overall quality. This seems to be in contradiction with MFA in Figure 6 (see below), in which OQJ at T12 was anti-correlated with treatment B. In practice, microvibration treatment (B treatment) was generally useful to improve the quality of a wine that was not yet ready for the market, but a 12-month period of vibration was too long.

### 3.4. Multifactor Analysis of the Effects of the Treatments after 12 Months of Storage

To better understand the treatment effect, the approach was to study the specific variability within a homogeneous time group. Thus, the analysis of a single time at the end of the storage (T12) made it possible to eliminate the effects on variance resulting from time, and to analyse the treatment effect only, after 12 months of storage. MFA (multiple factor analysis) was used as a data fusion method to understand the effect of all variables (from different analytical protocols) on the samples. For MFA, the dataset was divided into six different datasets; the variables from the sensory analysis constituted the first four datasets, classified according to the method of perception (visual, olfactory, taste and flavour, and overall quality). The last two datasets contained the volatile and phenolic compounds. Figure 6a shows the observation plot of time 12 obtained from MFA. Interestingly, in Figure 6a, wines stored for 12 months were clearly distinguished according to the different treatments undergone; the best treatments were A and C. Figure 6b–d show the variables plots for sensory analysis data, volatile compounds data, and phenolic compounds data, respectively, projected along the first two partial axes of the MFA. From these plots, it is possible to assess the correlations and anti-correlations between the parameters and those with the trends separating the samples; treatment B (application of microvibrations without damping) correlated with “woody”, “burning”, “sourness” and “unpleasant flavours”, but anti-correlated with the “overall quality judgment”. In addition, treatment B correlated with octanoic acid, ethyl ester (IX) and 2,4 di-tertbutyl phenol (XVIII) volatile compounds, and with the variables x.2, x.12 (*m*/*z* 579, procyanidin dimer-1), and x.24 phenolic compounds. Correlations between the concentration of volatile esters, including octanoic acid, ethyl ester (IX), and the application of vibration treatments versus control samples have been reported in the literature [8]. However, in this case it is more difficult to provide a general discussion for entire chemical classes, probably due to the low energy transmitted by the applied microvibrations. In fact, treatment A (no microvibrations, and with magnetic levitation) showed correlations with “clarity”, “woody flavour”, “astringency”, and “spicy flavour” for the sensory analysis variables, and with decanoic acid, ethyl ester (XIII) and propanoic acid, 2-hydroxy ethyl ester (VIII), for the volatile compounds, and with x.5 (*m*/*z* 591) and x.22 (*m*/*z* 465) for the phenolic compounds. Treatment C did not result in any particular correlation with the sensory analysis variables but was correlated with dodecanoic acid, ethyl ester (XVI) and 1-butanol, 3-methyl (V) for the volatile compounds, and x.3 and x.23 (syringic acid) for the phenolic compounds. Finally, treatment D was correlated with “colour intensity” and “bitterness” for the sensory analysis variables, ethyl acetate (II) and cyclopropane (IV) for volatile compounds, and x.6 (gallic acid) and x.25 (astilbin—isomer 1) for the phenolic compounds. Treatment D was able to reduce the negative influence of microvibrations applied in treatment B. Therefore, treatments that were shielded from prolonged microvibrations (except B, which was the treatment with the sound diffusion system) were more highly correlated with the volatile compounds.

## 4. Conclusions

It is well known that vibrations can modify and accelerate the process of ageing [18]. On the contrary, little is known on the long-term effects of microvibrations on high-quality wines, even when stored in optimal conditions. Our data were obtained by applying microvibrations and testing a potential microvibration damping device based on magnetic levitation over a storage period of one year for closed bottles with screw caps, a closure system that guarantees absolute airtightness.

The chemical analyses show that time had a much greater impact than treatment, as might be expected (PCA plots in Figure 2 and Figure 3). Only with sensory analysis was it possible to identify differences due to different treatments (particularly after 12 months of storage), because the microvibrations applied to the wine were associated with a low energy. The sensory analysis showed that the commercial wine was less appreciated by the panel at time 0 (when it was purchased from the winery, T0) than after 12 months of storage with different treatments. Regarding the effects of microvibrations, it was observed for the first time that the sensory panel liked the samples at T12 in which natural microvibrations were dampened with magnetic levitation (treatment A). The damping of microvibrations reduces the rate of wine evolution, keeping the wine quality similar to that of wine at T0. On the other hand, treatment with microvibration up to 6 months was useful for improving the quality of wine not yet ready for the market (OQ score = 6.30 for treatment B), whereas a 12-month period of microvibrations was too long (OQ score = 6.13).

In conclusion, this study shows the possibility of modulating the evolution of bottled wine by means of microvibrations or their damping to achieve the desired end result. In particular, it may be useful to dampen microvibrations for the preservation of fine wines (which are therefore already ready for trade), to maintain the high level of quality, while on the other hand, treatment with microvibrations may be useful to accelerate, in a positive sense, the evolution of wines that are not yet ready. However, an in-depth study is needed to identify the time and energy required to achieve the desired results.

## Figures and Tables

**Table 1:** List of descriptors and definitions used in the sensory analysis.

Descriptors	Definition
Visual evaluation	
Clarity	Absence of veiling and suspension in the wine
Colour tonality	Tonality of the colour red
Colour intensity	Intensity of the colour red
Olfactory evaluation	
Overall intensity	Total intensity of odour perceived through the nose
Red fruit	Strawberry, blueberries, raspberry, blackcurrant
Dried fruit	Strawberry jam, raisin, prune, fig
Undergrowth	Mushroom, wet wood, musk, fern
Spicy	Clove, black pepper, star anise, liquorice
Woody	Vanilla, oak, coffee
Cleanness	Absence of faults/taints odours
Off-odours	Presence of faults/taints odours
Gustatory evaluation	
Warmness	The sensation of alcohol (hot) in-mouth
Sweetness	Taste of sucrose
Sourness	Taste of acid solution
Saltiness	Taste of sodium chloride solution
Bitterness	Taste of caffeine solution
Astringency	Tactile sensation related to the dryness in the mouth
Cleanness	Absence of faults/taints flavours
Unpleasant flavours	Presence of faults/taints flavours
Overall quality judgment	An evaluation of the global quality of wine

## Data Availability

Acquired data are available upon request.

## Supplementary Materials

The following supporting information can be downloaded at: https://www.mdpi.com/article/10.3390/foods11182761/s1. Table S1: Tentative compounds identification by off-line LC-DAD-QqQ-MS indicates in table by (*). Compound assignment validated by standard injection indicates in table by (**); Table S2: Tentative compounds identification by GC × GC-TOF/MS analysis; Table S3: ANOVA results for factor Time on the phenolic compounds; Table S4: ANOVA results for factor Time on the volatile compounds.

## Abbreviations

ANOVA: analysis of variance; MFA: multifactor analysis; PCA: principal component analysis; GC × GC: comprehensive bidimensional gas chromatography; HPLC: high-performance liquid chromatography; MS: mass spectrometry; OQJ: overall quality judgment; HSD: honest significant difference; LC: liquid chromatography; DAD: diode array detector; FLD: fluorescence detector; QqQ: triple quadrupole mass detector; ESI: electrospray ionization; HS: headspace; SPME: solid-phase microextraction; DVB: divinylbenzene; CAR: carboxen; PDMS: polydimethylsiloxane; IS: internal standard.
