# Supplementary material for: Effects of Microvibrations and Their Damping on the Evolution of Pinot Noir Wine during Bottle Storage
Source: Foods. 2022 Sep 8;11(18):2761. doi: 10.3390/foods11182761 (PMC9498171; doi:10.3390/foods11182761)
Supplement: Supplementary file 1 [file foods-11-02761-s001.zip › foods-1878293-supplementary.pdf]

foods

MDPI

Supporting information

Effects of Microvibrations and Their Damping on the Evolution of Pinot Noir Wine during Bottle Storage

Simone Poggesi [1,2], Vakarė Merkytė [1,2], Edoardo Longo [1,2,*] and Emanuele Boselli [1,2]

[1] Oenolab, NOITechPark Alto Adige/Südtirol, Via A. Volta 13B, 39100 Bolzano, Italy
[2] Faculty of Science and Technology, Free University of Bozen-Bolzano, Piazza Università 5, 39100 Bolzano, Italy
* Correspondence: edoardo.longo@unibz.it; Tel.: +39-0471-017691

Citation: Poggesi, S.; Merkytė, V.; Longo, E.; Boselli, E. Effects of Microvibrations and Their Damping on the Evolution of Pinot Noir Wine during Bottle Storage. *Foods* **2022**, *11*, x. https://doi.org/10.3390/foods11182761

Received: 5 August 2022
Accepted: 31 August 2022
Published: 8 September 2022

Publisher's Note: MDPI stays neutral with regard to jurisdictional claims in published maps and institutional affiliations.

Copyright: © 2022 by the authors. Submitted for possible open access publication under the terms and conditions of the Creative Commons Attribution (CC BY) license (https://creativecommons.org/licenses/by/4.0/).

Table S1. Tentative compounds identification by off-line LC-DAD-QqQ-MS indicates in table by (*) .Compound assignment validated by standard injection indicates in table by (**) see https://mona.fiehnlab.ucdavis.edu/spectra/display/VF-NPL-QEHF028220, access on 29 July 2022.

Label	RT (min)	UV-Vis λ_{MAX} (nm)	Observed ESI- mass (*m*/*z*)			Tentative assignment	Ref.
x.1	5.0	264	179				
x.2	7.6	255	337	111		*p*-coumaroyl-quinic acid	[13]
x.3	10.5	288	269				
x.4	12.4	266	265	219			
x.5	16.0	310	591				
x.6	16.7	275	169			Gallic acid (*)	
x.7	20.5	281	591	156			
x.8	22.0	320	243				
x.9	23.8	284	368 (367)	305	193	Feruloyl-quinic acid	[14]
x.10	24.4	264,294	219	173	111	Shikimic acid, formic acid adduct	[15]
x.11	26.7	295,330	623	311		*Trans*-caftaric acid (*)	
x.12	29.1	280	640	577		Procyanidin dimer-1	
x.13	30.4		*175*	115		Ascorbic acid derivative	(**)
x.14	31.1	278	640	577		Procyanidin dimer-2	
x.16	32.6	280,310	295	163	149	Coutaric acid	[16]
x.17	33.2	280	579	352	289		
x.18	35.6	280	640	577		Procyanidin dimer-3	
x.19	36.4	295,325	179	135		*Trans*-caffeic acid (*)	
x.20	37.9	285	579	352	289		
x.21	41.1	285	582	382	319		
x.22	42.7	290	528	465			
x.23	43.3	275	197			Syringic acid (*)	
x.24	44.4	285,310	528				
x.25	49.4	290	449	512	303	Astilbin (isomer 1) (*)	
x.26	51.8	285	133	183	339		
x.27	52.5	292	449	512		Astilbin (isomer 2)	[17]

Table S2. Tentative compounds identification by GC × GC-TOF/MS analysis.

	Tentative identification	RT I° dim(min)	RT II° dim(sec)
I	Acetaldehyde	3.30	0.91
II	Ethyl acetate	4.67	1.74
III	n-propyl acetate	5.51	1.61
IV	Cyclopropane (NI)	12.97	0.88
V	1-butanol, 3-methyl-	17.27	1.10
VI	1-hexanol	23.65	1.74
VII	4-amino-1-butanol (NI)	23.65	1.31
VIII	Propanoic acid, 2-hydroxy-, ethyl ester	23.65	1.01
IX	Octanoic acid, ethyl ester	27.74	0.35
X	2-furancarboxaldehyde, 5-methyl-	32.99	0.99
XI	Isoamyl lactate	33.16	1.17
XII	Butyrolactone	35.62	1.30
XIII	Decanoic acid, ethyl ester	35.87	0.43
XIV	Butanedioic acid, diethyl ester	37.79	1.47
XV	3-buten-2-one, 4-(2,6,6-trimethyl-1-cyclohexen-1-yl)-	38.54	2.44
XVI	Dodecanoic acid, ethyl ester	43.09	0.07
XVII	Phenylethyl Alcohol	46.05	0.92
XVIII	2,4-di-tert-butylphenol	55.97	0.93
XIX	Hexadecanoic acid, ethyl ester	56.64	0.16

Table S3. ANOVA results for factor Time on the phenolic compounds.

	Phenolic compounds – Retention times (min)														
	**5.0**	**7.6**	**10.5**	**12.4**	**16.0**	**16.7**	**20.5**	**22.0**	**23.8**	**24.4**	**26.6**	**26.7**	**29.1**	**30.4**	**31.1**
T1	29721.45 b	116831.83 a	33346.17 b	91877.17 b	108933.00 c	1058927.17 b	61696.33 b	70209.25 a	35179.17 b	30172.64 c	28746.22 a	400089.83 c	288443.67 b	68946.17 c	55238.67 a
T3	25438.91 c	53523.25 c	48765.98 a	92757.25 b	83564.13 d	1078916.75 b	61234.88 b	30021.63 c	50261.38 a	33234.38 b	26929.25 b	410046.25 bc	291589.88 b	91615.88 b	56213.38 a
T6	32346.75 b	80612.38 bc	50518.13 a	85266.25 c	120062.25 b	1059315.25 b	63323.00 b	40927.00 b	48145.13 a	33959.25 b	29034.75 a	415211.13 b	274217.38 c	83690.25 b	44680.38 b
T12	55690.75 a	104603.88 ab	29866.99 c	290641.50 a	295549.13 a	2160025.38 a	68521.00 a	36303.15 bc	52218.88 a	68114.00 a	28452.30 a	782966.38 a	540286.38 a	375386.38 a	28984.22 c
Pr > F(Model)	<0.0001	0.00	<0.0001	<0.0001	<0.0001	<0.0001	<0.0001	<0.0001	<0.0001	<0.0001	0.00	<0.0001	<0.0001	<0.0001	<0.0001
Significant	Yes	Yes	Yes	Yes	Yes	Yes	Yes	Yes	Yes	Yes	Yes	Yes	Yes	Yes	Yes

	Phenolic compounds – Retention times (min)														
	**31.9**	**32.6**	**33.2**	**35.6**	**36.4**	**37.2**	**37.9**	**41.1**	**42.7**	**43.3**	**44.4**	**46.9**	**49.4**	**51.8**	**52.5**
T1	25023.82 c	390148.67 c	80475.81 a	54365.33 c	312371.17 c	134505.83 c	142955.83 c	53633.50 c	117525.83 a	178998.50 b	175618.83 c	48875.00 c	396962.50 b	31176.76 bc	31660.17 b
T3	46609.88 a	563175.75 b	28735.18 b	184114.88 b	349113.88 b	142106.63 b	166659.38 b	64891.38 b	108556.88 b	188675.25 b	212614.00 b	55583.00 b	403599.50 b	32726.75 b	33318.50 b
T6	46425.00 a	545341.25 b	47041.69 b	178006.38 b	293441.25 c	140758.00 bc	163162.25 b	61410.75 bc	60545.88 d	140397.75 c	176560.88 c	54156.88 bc	375450.50 c	27362.72 c	37359.38 b
T12	31481.07 b	742864.38 a	39705.75 b	285138.63 a	646516.88 a	298254.63 a	271463.75 a	123903.38 a	100478.25 c	252549.75 a	323034.88 a	129562.63 a	789546.75 a	71221.75 a	79880.63 a
Pr > F(Model)	<0.0001	<0.0001	0.00	<0.0001	<0.0001	<0.0001	<0.0001	<0.0001	<0.0001	<0.0001	<0.0001	<0.0001	<0.0001	<0.0001	<0.0001
Significant	Yes	Yes	Yes	Yes	Yes	Yes	Yes	Yes	Yes	Yes	Yes	Yes	Yes	Yes	Yes

a, ab, b, bc, c letters indicate the subgrouping created by the Tukey HSD test.

Table S4. ANOVA results for factor Time on the volatile compounds.

	I	II	III	IV	V	VI	VII	VIII	IX	X	XI	XII	XIII	XIV	XV	XVI	XVII	XVIII	XIX
T0	52.44 b	20.88 c	0.09 b	41.02 bc	104.66 c	0.00 b	0.61 b	10.31 d	6.32 b	1.45 cd	1.08 d	2.54 c	1.20 b	69.25 cd	0.92 cd	0.54 c	387.62 cd	111.22 ab	0.71 c
T1	171.43 a	101.39 b	8.97 a	547.09 a	1112.06 a	1.19 b	5.27 b	81.60 b	72.53 a	3.89 b	34.91 b	17.61 a	28.51 a	343.72 a	4.04 a	1.61 ab	1466.69 a	127.46 a	11.50 a
T3	22.17 b	23.92 c	1.15 b	18.20 c	234.69 c	0.65 b	2.82 b	18.90 cd	36.42 ab	0.90 d	2.76 d	2.63 c	13.97 ab	106.76 c	1.43 bc	0.90 bc	377.75 d	30.36 c	3.39 c
T6	41.60 b	64.28 bc	0.78 b	283.36 b	814.35 b	1.26 b	38.41 a	215.42 a	85.24 a	4.55 a	59.09 a	5.61 b	34.66 a	250.61 b	2.21 b	2.33 a	472.35 c	21.47 c	8.15 b
T12	48.26 b	540.95 a	8.37 a	427.11 ab	1015.97 ab	6.16 a	5.40 b	46.15 c	0.36 b	2.50 c	21.04 c	1.78 c	0.23 b	38.77 d	0.40 d	0.18 c	585.19 b	89.19 b	7.08 b
Pr > F(Model)	<0.0001	<0.0001	<0.0001	<0.0001	<0.0001	<0.0001	<0.0001	<0.0001	<0.0001	<0.0001	<0.0001	<0.0001	<0.0001	<0.0001	<0.0001	<0.0001	<0.0001	<0.0001	<0.0001
Significant	Yes	Yes	Yes	Yes	Yes	Yes	Yes	Yes	Yes	Yes	Yes	Yes	Yes	Yes	Yes	Yes	Yes	Yes	Yes

a, ab, b, bc, c letters indicate the subgrouping created by the Tukey HSD test.

References

13. Lee, Y.G.; Cho, J.Y.; Kim, C.M.; Lee, S.H.; Kim, W.S.; Jeon, T.I.; Park, K.H. Moon, J.H. Coumaroyl quinic acid derivatives and flavonoids from immature pear (Pyrus pyrifolia Nakai) fruit. *Food Sci. Biotechnol.* **2013**, *22*, 803–810. https://doi.org/10.1007/s10068-013-0148-z.
14. Kuhnert, N.; Jaiswal, R.; Matei, M.F.; Sovdat, T.; Deshpande, S. How to distinguish between feruloyl quinic acids and isoferuloyl quinic acids by liquid chromatography/tandem mass spectrometry. *Rapid Commun. Mass Spectrom.* **2010**, *24*, 1575–1582. https://doi.org/10.1002/rcm.4537.
15. Ivanova-Petropulos, V.; Naceva, Z.; Sándor, V.; Makszin, L.; Deutsch-Nagy, L.; Berkics, B.; Stafilov, T. Kilár, F. Fast determination of lactic, succinic, malic, tartaric, shikimic, and citric acids in red Vranec wines by CZE-ESI-QTOF-MS. *Electrophoresis* **2018**, *39*, 1597–1605. https://doi.org/10.1002/elps.201700492.
16. Baranowski, J.D.; Nagel, C.W. Isolation and identification of the hydroxycinnamic acid derivatives in white Riesling wine. *Am. J. Enol. Vitic.* **1981**, *32*, 5–13.
17. Fayad, S.; Le Scanff, M.; Waffo-Teguo, P.; Marchal, A. Understanding sweetness of dry wines: First evidence of astilbin isomers in red wines and quantitation in a one-century range of vintages. *Food Chem.* **2021**, *352*, 129293. https://doi.org/10.1016/j.foodchem.2021.129293.
